# β-Adrenergic receptor stimulation inhibits proarrhythmic alternans in postinfarction border zone cardiomyocytes: a computational analysis

**DOI:** 10.1152/ajpheart.00094.2017

**Published:** 2017-05-24

**Authors:** Jakub Tomek, Blanca Rodriguez, Gil Bub, Jordi Heijman

**Affiliations:** ^1^Life Sciences Interface Doctoral Training Centre, University of Oxford, Oxford, United Kingdom;; ^2^Department of Physiology, Anatomy and Genetics, British Heart Foundation Centre of Research Excellence, University of Oxford, Oxford, United Kingdom;; ^3^Department of Computer Science, British Heart Foundation Centre of Research Excellence, University of Oxford, Oxford, United Kingdom;; ^4^Department of Physiology, McGill University, Montreal, Quebec, Canada; and; ^5^Department of Cardiology, CARIM School for Cardiovascular Diseases, Maastricht University, Maastricht, The Netherlands

**Keywords:** alternans, β-adrenergic receptor stimulation, border zone, myocardial infarction, calcium, computational modeling

## Abstract

The border zone (BZ) of the viable myocardium adjacent to an infarct undergoes extensive autonomic and electrical remodeling and is prone to repolarization alternans-induced cardiac arrhythmias. BZ remodeling processes may promote or inhibit Ca^2+^ and/or repolarization alternans and may differentially affect ventricular arrhythmogenesis. Here, we used a detailed computational model of the canine ventricular cardiomyocyte to study the determinants of alternans in the BZ and their regulation by β-adrenergic receptor (β-AR) stimulation. The BZ model developed Ca^2+^ transient alternans at slower pacing cycle lengths than the control model, suggesting that the BZ may promote spatially heterogeneous alternans formation in an infarcted heart. β-AR stimulation abolished alternans. By evaluating all combinations of downstream β-AR stimulation targets, we identified both direct (via ryanodine receptor channels) and indirect [via sarcoplasmic reticulum (SR) Ca^2+^ load] modulation of SR Ca^2+^ release as critical determinants of Ca^2+^ transient alternans. These findings were confirmed in a human ventricular cardiomyocyte model. Cell-to-cell coupling indirectly modulated the likelihood of alternans by affecting the action potential upstroke, reducing the trigger for SR Ca^2+^ release in one-dimensional strand simulations. However, β-AR stimulation inhibited alternans in both single and multicellular simulations. Taken together, these data highlight a potential antiarrhythmic role of sympathetic hyperinnervation in the BZ by reducing the likelihood of alternans and provide new insights into the underlying mechanisms controlling Ca^2+^ transient and repolarization alternans.

**NEW & NOTEWORTHY** We integrated, for the first time, postmyocardial infarction electrical and autonomic remodeling in a detailed, validated computer model of β-adrenergic stimulation in ventricular cardiomyocytes. Here, we show that β-adrenergic stimulation inhibits alternans and provide novel insights into underlying mechanisms, adding to a recent controversy about pro-/antiarrhythmic effects of postmyocardial infarction hyperinnervation.

Listen to this article’s corresponding podcast at http://ajpheart.podbean.com/e/%CE%B2-ar-stimulation-and-alternans-in-border-zone-cardiomyocytes/.

## Abstract (precis)

We integrated, for the first time, postmyocardial infarction electrical and autonomic remodeling in a detailed, validated computer model of β-adrenergic stimulation in ventricular cardiomyocytes. Here, we show that β-adrenergic stimulation inhibits alternans and provide novel insights into underlying mechanisms, adding to a recent controversy about pro-/antiarrhythmic effects of postmyocardial infarction hyperinnervation.

electrical alternans, the beat-by-beat alternation of long and short repolarization durations, has been implicated in ventricular arrhythmogenesis and sudden cardiac death (SCD) (40, 49, 58). Patients with a history of myocardial infarction (MI) are at an increased risk of ventricular tachyarrhythmia and fibrillation (VT/VF) (28, 34, 51), and repolarization alternans is an independent predictor of SCD in MI patients (24, 60). Animal studies have shown that alternans can induce VT/VF via increased dispersion of refractoriness, e.g., promoting the formation of conduction block (5, 32, 35). Moreover, most post-MI arrhythmias originate in the infarct border zone (BZ), the zone of the viable myocardium adjacent to the infarct (53), which is also more prone to alternans than the normal myocardium (16).

Cellular mechanisms of alternans have been investigated in numerous experimental and computational studies (14, 15, 18, 31, 43, 46, 62), which have highlighted a complex interplay between different Ca^2+^ handling processes (57). In particular, a sarcoplasmic reticulum (SR) Ca^2+^ release-reuptake mismatch (8, 26, 62) or refractoriness of the type 2 ryanodine receptor (RyR2) channel, responsible for SR Ca^2+^ release (41), have been proposed to contribute to Ca^2+^ transient (CaT) alternans formation (Fig. 1*A*), which is then translated to repolarization alternans via the Na^+^/Ca^2+^ exchanger and other Ca^2+^-sensitive currents (31). However, remodeling in the BZ is complex, involving electrophysiological changes (42), cellular decoupling (47), and acute sympathetic denervation followed by sympathetic hyperinnervation (3, 12). The exact electrophysiological mechanisms determining how MI promotes alternans formation as well as the potential regulatory effects of sympathetic stimulation remain incompletely understood.

**Fig. 1.**
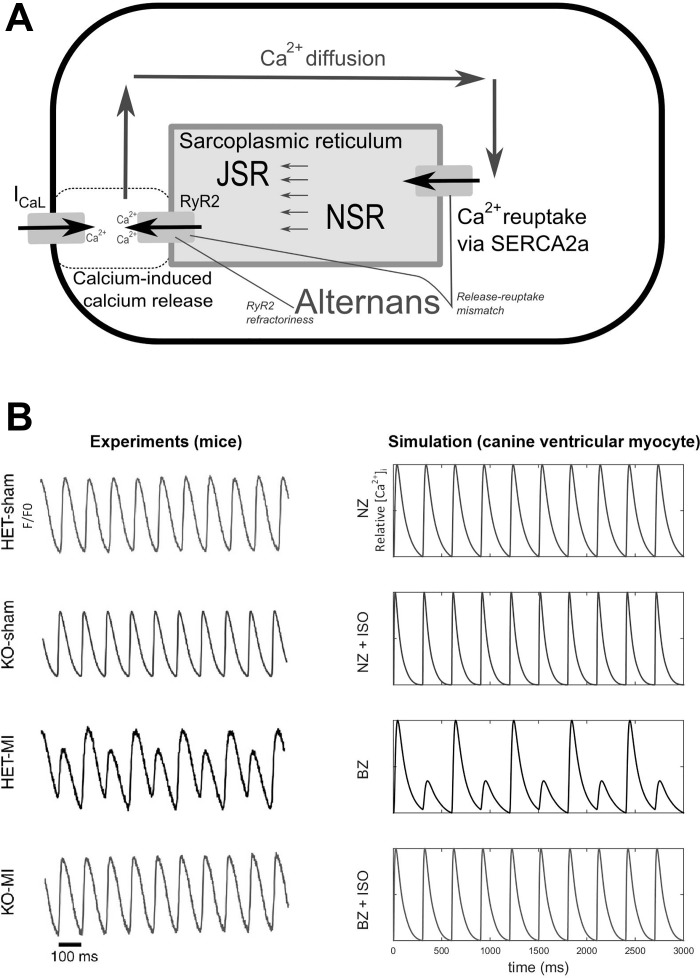
Mechanisms of alternans and effects of β-adrenergic receptor (β-AR) stimulation. *A*: schematic representation of Ca^2+^ handling in ventricular cardiomyocytes, where Ca^2+^ influx via L-type Ca^2+^ current (*I*_CaL_) triggers Ca^2+^ release from the sarcoplasmic reticulum (SR) via type 2 ryanodine receptors (RyR2). The Ca^2+^ then diffuses within the cell and is transported back to the SR via SERCA2a. Within the SR, two compartments are modeled: the junctional SR (JSR; the compartment containing RyR2s) and the network SR (NSR; containing SERCA2a). The predominant mechanisms of Ca^2+^ transient (CaT) alternans are based on RyR2 refractoriness (where the alternation of Ca^2+^ is driven by incomplete recovery from a sufficiently large release, inducing a small release only) or release-reuptake mismatch (where the alternans is due to reuptake insufficiency after a sufficiently large release). *B*: comparison of normalized CaT from the study of Gardner et al. (16) (*left* column) and our computational model (*right* column) under similar conditions. The experimental data come from mice, whereas the simulated traces are from a canine model, explaining the difference in duration and shape of CaTs. The heterozygous (HET)-sham trace is from myocardial infarction (MI)-free control mice; the knockout (KO)-sham trace is from MI-free mice with protein tyrosine phosphatase receptor-σ (PTPσ; an antireinnervation factor) knocked out, resulting in increased sympathetic innervation. The HET-MI trace is a trace from the denervated border zone (BZ), and the KO-MI trace is a trace from the hyperinnervated BZ. Hyperinnervation or simulated β-AR-stimulation with isoproterenol (ISO) abolished CaT alternans in the BZ.

It has been suggested that sympathetic hyperinnervation of the BZ is proarrhythmic (3, 30, 48, 54) and β-adrenoceptor (β-AR) blockers reduce microvolt T-wave alternans (27). On the other hand, mice with hyperinnervated infarct borders were less prone to repolarization and CaT alternans and were protected from arrhythmias compared with mice with denervated infarct borders (16). Recent human studies have also shown that lack of reinnervation after MI predicts future occurrence of SCD (2, 13). Similarly, β-AR agonists have been used to suppress repolarization alternans in some studies (14, 18, 26, 56) but increased alternans in others (36).

Detailed experimental characterization of the role of hyperinnervation in BZ arrhythmias is challenging because of the numerous distinct localized effects of post-MI remodeling and the diversity of species-specific (sub)cellular mechanisms controlling Ca^2+^ handling, repolarization alternans, and other proarrhythmic factors, all of which are modulated by β-AR stimulation. Computational models have been developed that can reproduce experimental electrophysiological characteristics, including repolarization and CaT alternans (15, 31, 62). Furthermore, these models allow perfect control over all parameters, ease of isolation of factors contributing to the behavior of the system, complete observability, and have been used to characterize subcellular mechanisms of alternans in more detail (15, 31, 46, 62). However, very few of these studies addressed BZ cardiomyocyte electrophysiology, and none incorporated regulation by β-AR stimulation.

Here, we hypothesized that β-AR stimulation would inhibit CaT and repolarization alternans in BZ cardiomyocytes and multicellular tissue. We characterized the interplay of BZ electrophysiological remodeling and β-AR stimulation on the likelihood of alternans by fusing an existing state-of-the-art computational model of β-AR stimulation in the canine ventricular cardiomyocyte (19) with a model of postinfarction electrophysiological remodeling in the BZ (23). Our results indicate that β-AR stimulation can indeed suppress alternans. Moreover, we identified a critical role for the regulation of SR Ca^2+^ release, both through activation of RyR2 channels and indirectly through regulation of SR Ca^2+^ load, in the suppression of alternans. The tools and insights resulting from this work facilitate a more comprehensive understanding of the complex pathophysiology involved in ventricular arrhythmogenesis post-MI.

## METHODS

### 

#### Model development.

We used the canine cardiomyocyte model by Heijman et al. (19), including the downstream effects of β-AR stimulation by arbitrary concentrations of the β-AR agonist isoproterenol (ISO) on L-type Ca^2+^ current (*I*_CaL_), phospholamban (PLB), slowly activating delayed rectifier K^+^ current (*I*_Ks_), RyR2, troponin I (TnI), fast Na^+^ current (*I*_Na_), Na^+^-K^+^ pump current (*I*_NaK_), and ultrarapid plateau K^+^ current (*I*_Kur_). A diagram of the model is provided in appendix a. The model can reproduce, among other things, ISO-dependent changes in cAMP levels, target phosphorylation, and electrophysiological properties, including action potential (AP) duration (APD) and CaT amplitude. We simplified the activation of RyR2 by β-AR stimulation in the model to facilitate its analysis while maintaining all relevant properties of the original model behavior. In particular, the number of parameter differences between phosphorylated and nonphosphorylated formulations has been reduced from four to three (activation constant, increased SR Ca^2+^ leak, and maximum release current multiplier); full details and motivation are provided in appendix b.

A BZ cell was modeled using the Heijman et al. model by applying BZ electrophysiological remodeling described in Hund et al. (23), changing the Ca^2+^/calmodulin-dependent protein kinase II (CaMKII) autophosphorylation rate (+425%), *I*_CaL_ (−36%), *I*_Na_ (−61%), transient outward K^+^ current (*I*_to_; full block), background Ca^2+^ current (*I*_Ca,b_; +33%), and time-independent inward rectifier K^+^ current (*I*_K1_; −40%). BZ cells maintain the original model of β-AR stimulation unless indicated otherwise.

To demonstrate the generality of the results, key experiments were repeated in the O’Hara-Rudy human ventricular cardiomyocyte model (38) by implementing the faster SR Ca^2+^ release due to β-AR stimulation-dependent RyR2 regulation to study the influence on alternans formation, as provided in appendix c.

#### Pacing protocols for alternans simulations.

Simulated fixed-rate pacing was used to study the formation of repolarization alternans and underlying CaT alternans at a given basic pacing cycle length (BCL). The pacing stimulus was a 1-ms-long pulse at −80 pA/pF, because in less excitable BZ cells the duration of 0.5 ms common in in silico studies failed to trigger an AP at fast pacing rates. Amplitude of repolarization alternans was defined as maximum APD − minimum APD of the last 10 APDs of each simulation. The alternans ratio of APD or CaT amplitude was defined as 1 – minimum/maximum, reflecting the minimum and maximum values of two successive beats, respectively, in line with previous experimental studies (14, 18). A simulation was labeled as manifesting alternans when the amplitude of its APD alternans exceeded 3 ms or when the alternans ratio was larger than 0.02. All data were obtained in a quasistable state (defined in appendix d). To determine the impact of downstream effects of β-AR stimulation separately (used in *Role of SR Ca^2+^ release modulation in abolishing CaT alternans* and *Implications for arrhythmogenesis post-MI*), the signaling cascade of the model was turned off and the phosphorylation levels of various sites were manually clamped to either 0 (nonphosphorylated) or to 1 (fully phosphorylated). The model was modified to similarly allow isolating the distinct effects of RyR2 activation via β-AR stimulation.

#### Model implementation.

Single cell simulations were performed using the previously published MATLAB (The Mathworks, Natick, MA) code of the Heijman et al. model (19) using the ode15s solver. Fiber simulations were performed using the Myokit software package (7). The OpenCL-based support for parallel computation available in Myokit was used for multicellular simulations with a fixed time step of 0.0025 ms. The model code is available as Supplemental Material (Supplemental Material for this article is available at the *American Journal of Physiology-Heart and Circulatory Physiology* website.).

## RESULTS

### 

#### β-AR stimulation abolishes APD and CaT alternans.

**Fig. 2.**
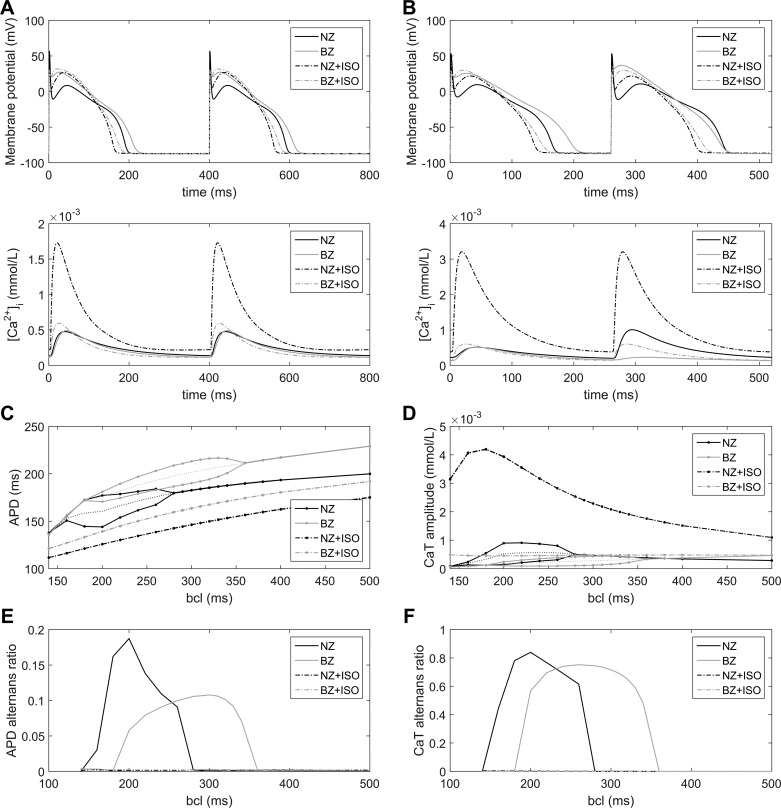
Effect of β-AR stimulation on the rate dependence of alternans. *A*: membrane potential and Ca^2+^ transients of the following four model configurations at a basic cycle length (BCL) of 400 ms: NZ cell, BZ cell, β-AR-stimulated (1.0 µmol/l ISO) NZ cell (NZ + ISO), and β-AR-stimulated BZ cell (BZ + ISO). *B*: analogous plot at a BCL of 260 ms, showing action potential (AP) duration (APD) and CaT alternans in NZ and BZ cases in the absence of β-AR stimulation. *C* and *D*: APD and CaT rate dependence curves for the four simulated configurations. Bifurcation in NZ and BZ curves indicates the occurrence of alternans. The dotted lines represent the mean of minimum and maximum APD during alternans. *E* and *F*: alternans ratio for APD and CaT alternans in the four groups.

Our model replicated characteristics of the experimentally observed alternans formation and its relationship to β-AR stimulation (16), as shown in Fig. 1*B*. In particular, it showed that denervated BZ cardiomyocytes may manifest CaT alternans at slower pacing frequencies than myocytes from noninfarcted normal zone (NZ) myocardium and that this increased susceptibility is not present in hyperinnervated BZ cardiomyocytes. We simulated the APD rate dependence and alternans occurrence at various BCLs in NZ and BZ with and without maximal β-AR stimulation (1.0 µmol/l ISO; Fig. 2). The maximum slope of the APD rate dependence curve (Fig. 2*C*) never exceeded 1.0, indicating that the APD alternans formation under these conditions is not caused by steep restitution. In agreement, an underlying CaT alternans was observed in all cases of APD alternans. BZ cells manifested alternans at slower pacing frequencies than NZ cells (alternans onset at a BCL of 340 vs. 280 ms; Fig. 2*C*), as was also evident from the rate dependence of alternans ratio (Fig. 2, *E* and *F*). In contrast, the magnitude of maximal APD and CaT alternans was slightly smaller in BZ (change in APD: 22 ms, APD alternans ratio: 0.11; change in CaT: 291 nmol/l, CaT alternans ratio: 0.75) compared with NZ (change in APD: 33 ms, APD alternans ratio: 0.19; change in CaT: 751 nmol/l, CaT alternans ratio: 0.83) models. The β-AR stimulation abolished both APD and CaT alternans in NZ and BZ models at all BCLs.

#### Electrophysiological mechanisms underlying β-AR stimulation-induced alternans inhibition.

To discern which of the eight downstream targets of β-AR signaling incorporated in the model are responsible for the abolishment of CaT alternans, we analyzed CaT alternans formation at bcl of 260 ms for all 256 combinations of either dephosphorylated or fully phosphorylated targets. The strongest alternans-abolishing effect was achieved with the simulated phosphorylation of RyR2, preventing CaT alternans in all 128 combinations involving this downstream effect (not shown).

**Fig. 3.**
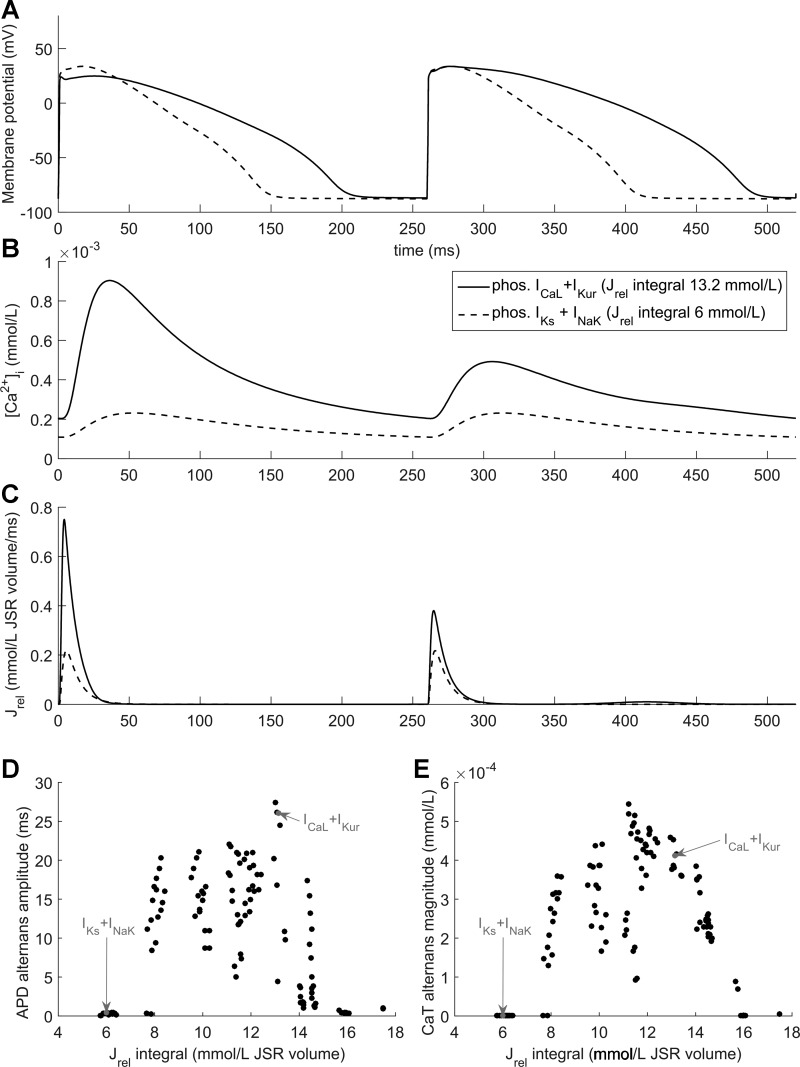
Role of SR Ca^2+^ release magnitude in alternans. *A−C*: AP (*A*), cytosolic Ca^2+^ concentration (*B*), and SR Ca^2+^ release flux (*J*_rel_; *C*) for two combinations of downstream β-AR stimulation effects (phosphorylated *I*_CaL_ and *I*_Kur_, solid line; phosphorylated *I*_Ks_ and *I*_NaK_, dashed line) at a BCL of 260 ms. *D* and *E*: magnitude of APD (*D*) and CaT (*E*) alternans for all 2^7^ = 128 different combinations of downstream effects of β-AR without RyR2 activation versus the integral of *J*_rel_ over two consecutive APs; the two conditions shown in *A–C* are indicated with arrows.

There are 128 (2^7^) possible combinations of β-AR signaling phosphorylation targets in the absence of RyR2 phosphorylation, 88 of which showed alternans at a BCL of 260 ms (e.g., phosphorylated *I*_CaL _+ *I*_Kur_) and 40 of which did not (e.g., phosphorylated *I*_Ks _+ *I*_NaK_; Fig. 3, *A*–*C*). Figure 3, *D* and *E*, shows the amount of APD and CaT alternans as a function of the total amount of Ca^2+^ released from the SR during two BCLs (2 BCLs were used instead of a single beat to enable comparisons between cells in alternans and without) for each of these 128 combinations. Combinations without RyR2 phosphorylation that did not manifest APD, and CaT alternans could be divided into two major groups. The first group involved those combinations of downstream effects that diminished SR Ca^2+^ release (e.g., phosphorylation of *I*_NaK_, which increases its activity and lowers Na^+^ and Ca^2+^ levels). Alternans formation in the family of models used in this study is predominantly due to a mismatch between Ca^2+^ release and Ca^2+^ reuptake at rapid pacing rates, causing the next Ca^2+^ release to be smaller, which, in turn, makes the next release larger, etc. (31, 62). However, when Ca^2+^ release is sufficiently diminished, this mismatch does not occur, limiting the formation of CaT alternans (Fig. 3).

**Fig. 4.**
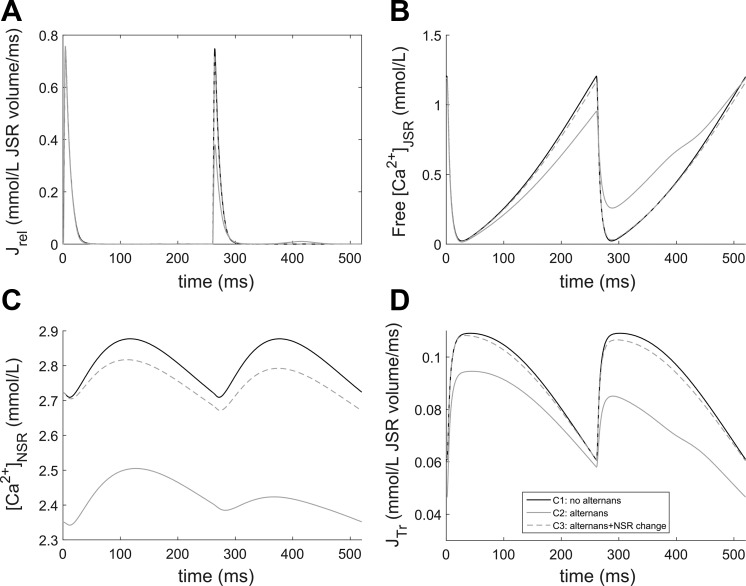
Effect of SR Ca^2+^ load on alternans. SR Ca^2+^ release flux (*A*), JSR (*B*) and NSR (*C*) Ca^2+^ concentrations, and Ca^2+^ transport between the NSR and JSR (*D*) are shown for the following three simulations: C1 (a combination of β-AR-stimulated downstream effects with high release and no alternans), C2 (a combination of β-AR-stimulated downstream effects with relatively high release, manifesting alternans), and C3 (C2 with NSR Ca^2+^ content changed to that of C1 at the beginning of the plotted period).

The second major group of β-AR stimulation-dependent effects preventing the formation of alternans was the group with the largest SR Ca^2+^ release and a fast Ca^2+^ reuptake. This group involved increases in *I*_CaL_ and SERCA2a activity (via reduced PLB-mediated inhibition on phosphorylation), optionally with other β-AR-mediated effects that do not decrease SR Ca^2+^ release. In this case, the overall SR Ca^2+^ load increased and the junctional SR (JSR) emptied almost completely during releases. In this setting, increased loading of the network SR (NSR) increased Ca^2+^ diffusion from the NSR to JSR, preventing CaT alternans, since even after a large Ca^2+^ release, the reuptake and NSR-to-JSR-transport mechanisms were fast enough to maintain a normal subsequent release. To illustrate this effect, we considered two combinations of downstream effects: C1, free of alternans due to large Ca^2+^ release (simulated phosphorylation of *I*_CaL _+ PLB + TnI + *I*_Na_), and C2, manifesting alternans (phosphorylation of *I*_CaL _+ *I*_Kur_), with the large Ca^2+^ release in C2 closely matching the Ca^2+^ releases of C1. The Ca^2+^ handling properties of these variants during two APs at a BCL of 260 ms are shown in Fig. 4. In C1 and C2, the first Ca^2+^ release was nearly identical despite the different NSR contents, which affected the speed of JSR transport (*J*_tr_) and thus JSR refilling. Because in the C2 model the JSR was not refilled sufficiently after the first release, and given that the SR Ca^2+^ load-release relationship is steep, the next Ca^2+^ release is comparatively small and a relatively large amount of Ca^2+^ remains in the JSR. More Ca^2+^ is then added to the JSR during and after the second release, but the process is limited by the high level of Ca^2+^ in the JSR, reducing the concentration gradient between NSR and JSR (Fig. 4). As a third case (C3), we considered a model with the same parameters as C2 but which had its NSR contents artificially changed to the value of C1 at the start of the larger SR Ca^2+^ release flux (*J*_rel_) at the beginning of the shown interval. In this case, the second release was almost identical to the first release, supporting the importance of NSR loading (promoted by a high Ca^2+^ reuptake rate) in alternans prevention.^1^

#### Biophysical RyR2 properties regulating CaT alternans.

With simulated downstream effects of β-AR stimulation on RyR2, alternans was never present, independent of the integrated *J*_rel_ over two APs (ranging from 5.2 to 16.4 mmol/l). Thus, the total amount of Ca^2+^ released is not the key mechanism behind the abolishment of alternans under these conditions. To discern the mechanism behind the antialternans effect of RyR2 activation, we isolated and separately simulated the three downstream effects of β-AR stimulation on RyR2 biophysical properties. Under maximal β-AR stimulation, the time constant of RyR2 activation and deactivation was reduced to ~21% of the original value, the SR Ca^2+^ release magnitude was reduced to 65%, and the JSR Ca^2+^ leak was increased as in the original model. All effects of β-AR stimulation other than RyR2 activation were disabled, unless stated otherwise, to isolate the effects of altered RyR2 dynamics from other downstream effects.

We simulated all eight possible combinations of the three β-AR-stimulation-induced changes to release dynamics (Table 1). Most combinations of RyR2 activation changes abolished CaT alternans, but the mechanisms involved turned out to be distinct. To better understand the impact of the RyR2 activation changes on alternans formation, we stimulated a BZ cell without β-AR stimulation at a BCL of 260 ms and acutely introduced each of the three single RyR2 activation changes individually, allowing us to observe the immediate effect of these changes, without potentially confounding effects on intracellular ionic concentration or SR Ca^2+^ load.

**Table 1.** Border zone model with individual effects of RyR2 activation via β-AR stimulation (with all other β-AR stimulation downstream effects disabled)

RyR2_τ Change	RyR2_Amp Change	RyR2_leak Increase	APD Alternans Amplitude, ms	Release Integral, mmol/l JSR volume
BZ model without β-AR stimulation			20.13	8.62
True	True	True	0.32	7.69
True	True	False	0.35	8.00
True	False	True	0.19	8.02
True	False	False	0.39	8.32
False	True	True	0.49	8.18
False	True	False	0.37	8.54
False	False	True	14.05	8.43

The sarcoplasmic reticulum (SR) Ca^2+^ release integral is a sum of the Ca^2+^-induced Ca^2+^ release flux and the junctional SR (JSR) leak over two beats. True/false indicates whether or not the given effect is active or not. RyR2, type 2 ryanodine receptor; β-AR, β-adrenergic receptor; RyR2_τ, RyR2 time constant; RyR2_Amp, RyR2 amplitude of SR Ca^2+^ release; APD, action potential duration.

**Fig. 5.**
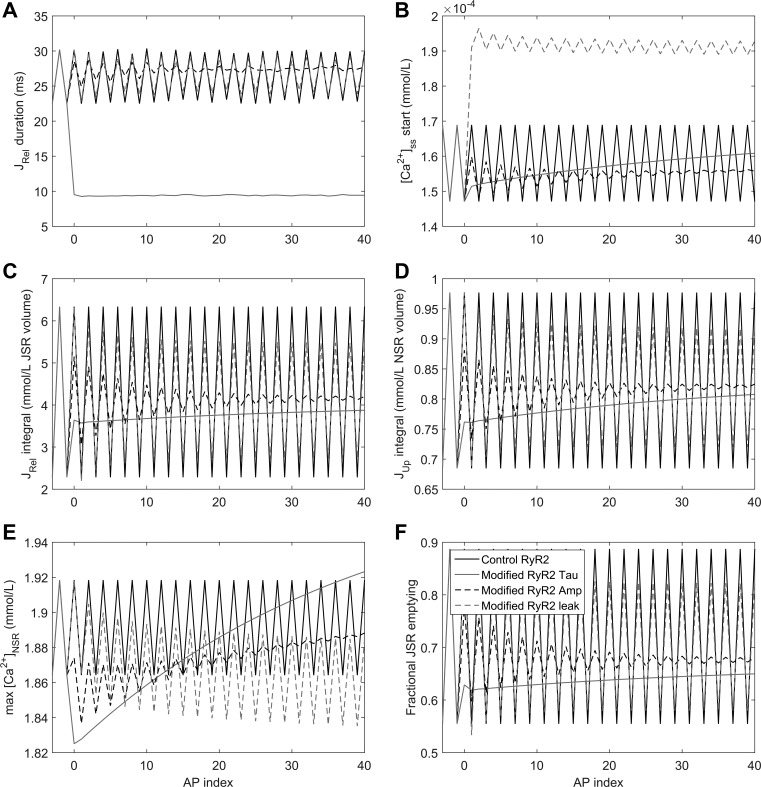
Effect of acute introduction of single RyR2 activation changes on alternans. Release duration (*A*), diastolic Ca^2+^ concentration in the subspace (*B*), SR Ca^2+^ release integral (*C*), SR Ca^2+^ uptake integral (*D*), maximal NSR Ca^2+^ content (*E*), and fractional JSR emptying (*F*) are shown as a function of the AP index after stabilization (APs of 2,437–2,480 are shown from a sequence of 2,500 APs, with RyR2 activation from an AP index of 0, corresponding to an AP of 2,440). The model was paced at a BCL of 260 ms under control conditions or with selective changes to RyR2 time constants (τ), RyR2 amplitude of SR Ca^2+^ release (Amp), or JSR Ca^2+^ leak.

A faster time constant (RyR2_τ) produced an immediate decrease in the duration of SR Ca^2+^ release (measured as the duration of *J*_rel_ > 0.025; Fig. 5*A*), accompanied by a small decrease in the integrated SR Ca^2+^ release (Fig. 5*C*), which could also be observed as slightly reduced fractional emptying of the JSR (Fig. 5*F*). With reduced RyR2_τ, SR Ca^2+^ uptake exceeds *J*_rel_ (Fig. 5*D*), resulting in steeply increasing NSR loading (Fig. 5*E*). There are two reasons for this phenomenon. The first reason is that faster SR Ca^2+^ release led to comparatively higher Ca^2+^ gradients between the cytosol and NSR, increasing the efficiency of reuptake via SERCA2a. Second, the faster upstroke of Ca^2+^ release meant that the main Ca^2+^ load was released earlier than with baseline Ca^2+^ release dynamics, giving more time for Ca^2+^ reuptake. Both these factors thus increase reuptake, improving JSR refilling and reducing SR Ca^2+^ release refractoriness.

The most notable effect of increased JSR Ca^2+^ leak was an increased Ca^2+^ concentration in the subspace between RyR2 and L-type Ca^2+^ channels (Fig. 5*B*). Because of a reduced driving force, the increased leak then acted as a dampener, limiting the systolic SR Ca^2+^ release (Fig. 5*C*), preventing overt emptying of the JSR and providing more Ca^2+^ for the next release (Fig. 5*G*), thus gradually reducing alternans.

Finally, the antialternans effect of decreasing the RyR2-mediated amplitude of SR Ca^2+^ release (RyR2_Amp) was chiefly due to limiting the amount of Ca^2+^ released, which acted first during the large SR Ca^2+^ release, thereby providing more Ca^2+^ for the next release, similar to an increase in SR Ca^2+^ leak.

**Fig. 6.**
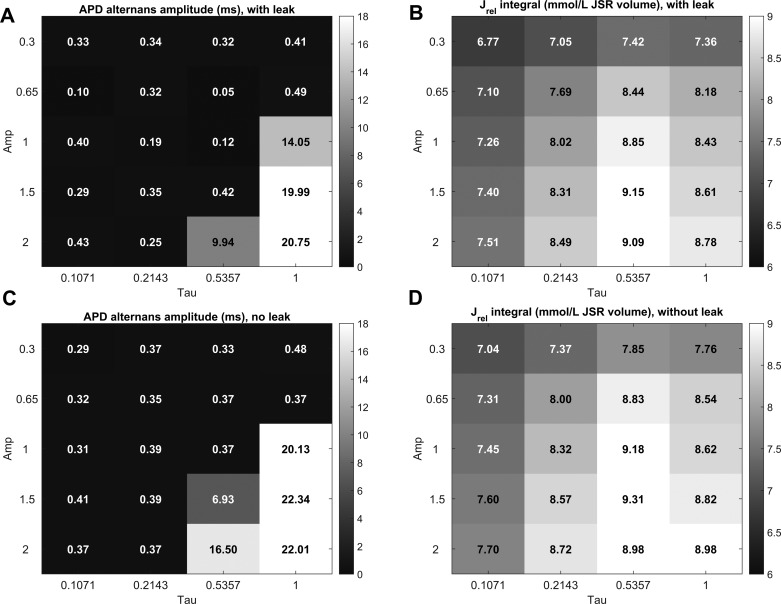
Sensitivity analysis of the effects of altered RyR2 gating on alternans. Alternans amplitude is shown for different combinations of τ and Amp properties of RyR2, either with increased JSR leak (*A*) or without (*B*). Similarly, *B* and *D* show the integral of SR Ca^2+^ release over two consecutive APs with or without the presence of increased JSR leak, respectively.

To investigate the robustness of these results, we evaluated a range of RyR2_Amp and RyR2_τ values, measuring the APD alternans amplitude and the integral of SR Ca^2+^ released over two consecutive APs (Fig. 6). A wide range of parameter combinations could prevent alternans, particularly those where RyR2_τ was reduced. As shown in the last column of Fig. 6, a decrease in RyR2_Amp could prevent alternans only by directly decreasing the amount of Ca^2+^ released, i.e., producing a leftward shift to the nonalternans region shown in Fig. 3. A decrease in RyR2_τ, on the other hand, prevented alternans even in cases when the amount of SR Ca^2+^ released was higher than the 8.62 mmol/l from the control case (relative RyR2_Amp = RyR2_τ = 1 and no additional leak), suggesting that the accelerated SR Ca^2+^ release dynamics are the most important factor limiting alternans.

We confirmed the generality of this result by testing the effect of decreasing the SR Ca^2+^ release time constant on alternans formation in another computational model, the state-of-the-art human ventricular cardiomyocyte model by O’Hara et al. (38). The results, described in detail in appendix c, are in agreement with our main results, confirming that accelerated RyR2 kinetics are a potent attenuator of alternans.

#### Cell-to-cell coupling modulates alternans incidence.

**Fig. 7.**
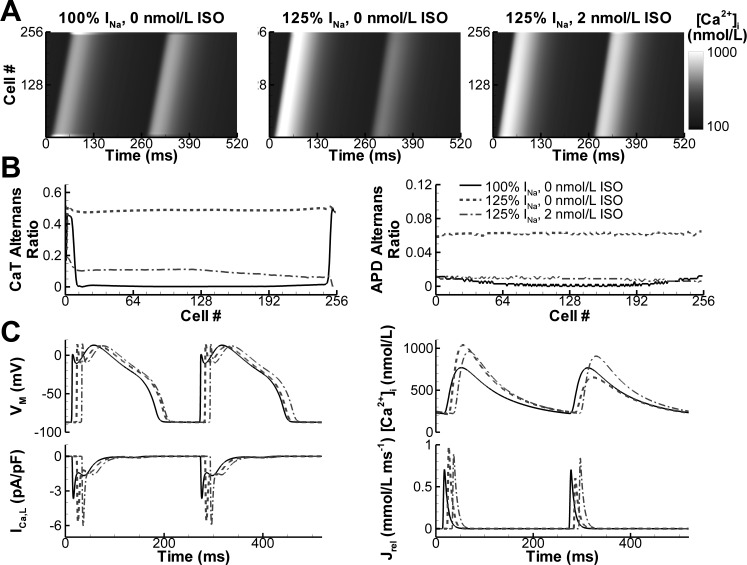
Effects of β-AR stimulation on alternans in one-dimensional strand simulations. *A*: CaTs along a 256-cell strand at a BCL of 260 ms for the control model, model with increased *I*_Na_, and model with increased *I*_Na_ and homogenous β-AR stimulation. *B*: CaT and APD alternans ratio along the strand for the three model versions. *C*: mechanism underlying the absence of alternans in the control model (solid line) compared with the model with increased *I*_Na_ (dashed line) and the abolishment of alternans by β-AR stimulation (dashed-dotted line) as evident from AP, CaT, *I*_CaL_, and SR Ca^2+^ release flux. Note the smaller *I*_CaL_ under control conditions. Slightly different positions along the strand are shown for the three conditions to enhance the presentation.

To analyze the effects of electrotonic coupling between cardiomyocytes on alternans generation, we investigated alternans incidence and its modulation by β-AR stimulation in one-dimensional strand simulations (10). Under conditions in which a single NZ cardiomyocyte model exhibited alternans (BCL = 260 ms, no β-AR stimulation), a 256-cell strand of electrotonically coupled NZ cardiomyocytes (conduction velocity: 55 cm/s) only showed CaT alternans at the very beginning and end of the strand (Fig. 7*A*, *left*), which was not sufficient to produce APD alternans (Fig. 7*B*, *right*). Increasing the Na^+^ current amplitude by 25% resulted in robust CaT and APD alternans throughout the strand, which could be inhibited by simulated β-AR stimulation (Fig. 7, *A* and *B*). We investigated the mechanism underlying the modulation of alternans by cell-to-cell coupling and found that the reduced AP upstroke due to the electrotonic load resulted in a smaller peak *I*_CaL_, which triggered a smaller SR Ca^2+^ release (Fig. 7*C*). This reduced SR Ca^2+^ release was indeed within the non-alternans region shown in Fig. 3. On the other hand, β-AR stimulation reduced alternans in the strand predominantly via the direct effects on RyR2 gating, since simulated RyR2 phosphorylation alone similarly abolished alternans (not shown).

#### Differences in β-adrenergic sensitivity between NZ and BZ cardiomyocyte models.

**Fig. 8.**
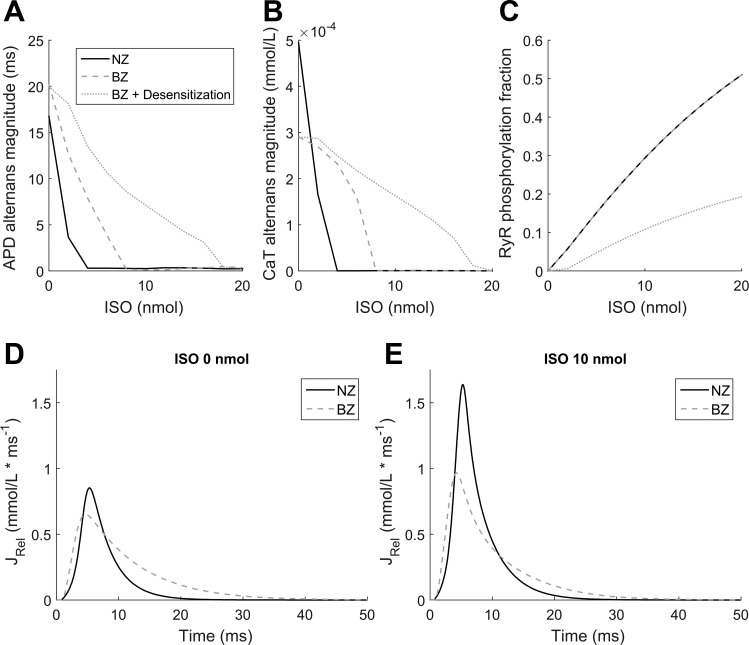
Differences in β-adrenergic sensitivity between the NZ and BZ. APD (*A*) and CaT (*B*) alternans magnitude and fraction of phosphorylated RyR2 (*C*) versus ISO concentration are shown at a BCL of 260 ms for a NZ cell, BZ cell, and a BZ cell with desensitized β-AR (−25% expression). *D* and *E*: comparison of SR Ca^2+^ release flux at a BCL of 500 ms between the NZ and BZ in the absence (*D*) or presence (*E*) of β-adrenergic stimulation (10 nmol/l ISO).

As maximal β-AR stimulation abolished alternans in both NZ and BZ, we subsequently tested the effects of different ISO concentrations in each model at a BCL of 260 ms, a frequency at which both cell types manifested alternans. The BZ model required almost double the dose of ISO of NZ cells to eliminate APD and CaT alternans (Fig. 8, *A* and *B*) and more than four times that of NZ cells when taking into account the remodeling of β-AR expression (−25%) that has been reported to occur in BZ tissue (52). We identified two major contributors to the reduced sensitivity of the BZ model. First, in the BZ with reduced β-AR expression, phosphorylation of RyR2 is lower than that of the NZ (Fig. 8*C*), which reduces the RyR2 phosphorylation-dependent inhibition of alternans. Second, our sensitivity analyses (Fig. 6) have shown that faster time constants (i.e., a lower RyR2_τ parameter) inhibit alternans formation, but the baseline BZ model has a markedly longer duration of SR Ca^2+^ release than the NZ model (Fig. 8*D*), thereby making the BZ more vulnerable to alternans. β-AR stimulation reduces the SR Ca^2+^ release duration (Fig. 8*E*), but more β-AR stimulation is needed to overcome the baseline characteristics of the BZ model that promote alternans.

## DISCUSSION

We used computational modeling to provide insights into the modulation of repolarization and CaT alternans by β-AR stimulation. We found that β-AR stimulation abolishes both forms of alternans in NZ as well as BZ models, representing the surviving myocardium surrounding an infarct with increased susceptibility to alternans. Repolarization alternans was primarily driven by beat-to-beat fluctuations in Ca^2+^ cycling. Both direct modulation of RyR2 properties by β-AR stimulation as well as indirect modulation of SR Ca^2+^ release through the effects of other β-AR targets can abolish CaT alternans and thereby repolarization alternans. Although cell-to-cell coupling influenced the occurrence of alternans, β-AR stimulation was able to inhibit alternans formation both in single cell and multicellular simulations. Together, these findings suggest that BZ hyperinnervation may antagonize the development of proarrhythmic repolarization alternans.

### 

#### Comparison with previous work.

Numerous studies have investigated the subcellular determinants of CaT and repolarization alternans (15, 31, 46, 62). In general, these studies have identified a critical role for SR Ca^2+^ cycling properties in CaT alternans development, focusing either on the refractoriness of SR Ca^2+^ release channels or slow Ca^2+^ reuptake resulting in a release-reuptake mismatch. The results presented here are in agreement with these findings and, for the first time, use computational modeling to study the effects of β-AR stimulation on CaT alternans. We have previously developed a model incorporating the entire β-AR signaling cascade from receptor to target phosphorylation that can reproduce the major effects of β-AR stimulation including changes in AP morphology and APD, increased CaT amplitude, and faster CaT decay (19). The antialternans effect of β-AR stimulation observed in the present study is in agreement with a number of experimental studies in both atrial and ventricular cardiomyocytes of a variety of species (14, 18). However, given the complex downstream effects of β-AR stimulation, affecting numerous ion channels, Ca^2+^ handling proteins, and signaling cascades, it is experimentally challenging to identify the exact molecular mechanisms involved. As such, we used the perfect observability and control provided by computational modeling and provided novel evidence that there are multiple combinations of downstream effects that may abolish CaT alternans. Moreover, we identified modulation of SR Ca^2+^ release properties, either directly or indirectly, as the central element of the antialternans effects of β-AR stimulation (discussed below).

Arrhythmias are an intrinsically multicellular phenomenon, and the effects of sympathetic stimulation on alternans in tissue appear more complex. Huang et al. (22) found that left stellate ganglion stimulation promoted repolarization alternans in vivo, but this correlated strongly with steep restitution properties, suggesting a Ca^2+^-independent mechanism that may also be affected by altered cardiac metabolism during long-term sympathetic stimulation. Spatially heterogeneous alternans is significantly more proarrhythmic than spatially homogeneous (concordant) alternans because of the presence of steep spatial repolarization gradients (57). Restoring sympathetic innervation in the infarct BZ reduced CaT alternans, repolarization dispersion, and arrhythmogenesis in mice (16). Hammer et al. (18) showed that alternans susceptibility in mice was modulated by gap junction coupling and that ISO reduced alternans amplitude in uncoupled tissue but had no effect in control tissue. Our simulations similarly showed that electrotonic interactions can modulate the occurrence of alternans, and we determined this to be due to modulation of Ca^2+^-induced Ca^2+^ release via reduced *I*_Ca,L_. In addition, local changes in extracellular ion concentrations in tissue may affect electrophysiological properties. For example, accumulation of extracellular K^+^ may contribute to changes in resting membrane potential and APD, potentially explaining why APD is prolonged in isolated BZ cardiomyocytes and computational models but is shorter than NZ APD in multicellular preparations. All our multicellular simulations produced spatially concordant alternans, and β-AR stimulation reduced alternans both in single cell and multicellular simulations, independent of extracellular K^+^ levels (not shown).

#### Role of SR Ca^2+^ release modulation in abolishing CaT alternans.

We identified a central role for the modulation of SR calcium release properties in the inhibitory effects of β-AR stimulation on CaT alternans. In particular, either direct or indirect modulation of SR Ca^2+^ release via RyR2 or other downstream targets of β-AR stimulation can inhibit alternans formation.

The indirect effects inhibited CaT alternans by either lowering total SR Ca^2+^ release (Fig. 3), which reduces the emptying of the JSR and lowers the beat-to-beat release-reuptake mismatch, or by significantly increasing SR Ca^2+^ release. In eight specific combinations of phosphorylation effects, the SR Ca^2+^ release was strong enough to release the entire contents of the JSR, leading to an abrupt drop in SR release to zero. This type of release acted against alternans, as the rapid release of large amounts of Ca^2+^ stimulated SERCA2a to rapidly reuptake Ca^2+^. However, as the release ended abruptly because of emptying of the JSR, the total amount of Ca^2+^ released was lower than that of a normal-shaped, smoothly decaying SR Ca^2+^ release with an identical initial segment. This phenomenon puts an upper bound on the SR Ca^2+^ release for a given beat and aids the system in preventing the release-reuptake mismatch. A similarly shaped release current has recently been observed in a study using in vivo human data and computational modeling to investigate the formation of so-called eye-type alternans (alternans that opens as the pacing frequency increases and then disappears with a further increase in frequency) (62). There, this Ca^2+^ release phenotype was detected in a subpopulation of virtual cardiomyocytes with upregulated *I*_Ca,L_, where it occurred in the closed phase of eye-type alternans at high frequencies. Thus, this mechanism could be relevant for explaining why alternans may disappear as the pacing frequency is increased.

RyR2 can be phosphorylated on Ser^2030^ and Ser^2809^ by PKA and on Ser^2815^ by CaMKII in response to sympathetic stimulation. The exact pathways and phosphorylation sites mediating β-AR stimulation-dependent modulation of RyR2 and their relevance for cardiac (patho)physiology remain controversial (11). However, despite the lack of understanding of the exact mechanism, β-AR stimulation appears to sensitize RyR2 gating, increasing Ca^2+^ leak from the SR and producing faster and shorter Ca^2+^-induced Ca^2+^ release transients during systole (17, 50, 61). Our model of RyR2 activation by β-AR stimulation replicates these key macroscopic features of SR Ca^2+^ release under β-AR stimulation. We were able to link the accelerated RyR2 opening to alternans attenuation via improvement in the SR Ca^2+^ reuptake. Of note, this mechanism can be independent from changes in RyR2 refractoriness previously reported to modulate CaT alternans (41). However, the earlier-terminated SR release could also provide more time for RyR2 channels to recover from refractoriness, making SR Ca^2+^ release kinetics also relevant for RyR2 refractoriness-driven alternans.

To ensure that the observed effects did not solely apply to the particular formulation of SR Ca^2+^ handling in the canine ventricular cardiomyocyte model, we incorporated the effects of β-AR stimulation on SR Ca^2+^ release in the O’Hara-Rudy model of the human ventricular cardiomyocyte (38), similarly observing the attenuation of alternans. Furthermore, these effects are consistent with experimental data showing that caffeine-induced sensitization of RyR2 reduces CaT alternans in rabbit hearts (56). A secondary antialternans effect of the RyR2 modulation by β-AR that we identified was increased SR Ca^2+^ leak, which increases subspace Ca^2+^ concentrations, reducing the gradient between the JSR and cytosol, limiting the possibility of an overly large release. An interesting property of such dampening of CaT alternans by increased SR Ca^2+^ leak is that it is dynamic: the larger the SR Ca^2+^ load, the more Ca^2+^ leaks out and thus the more the next release is limited.

#### Implications for arrhythmogenesis post-MI.

BZ cardiomyocytes are more prone to alternans, even at relatively slow pacing frequencies (Fig. 2), which would further promote proarrhythmic spatially discordant alternans. The pro- or antiarrhythmic effects of sympathetic hyperinnervation in the BZ remain a topic of debate. Studies artificially upregulating the degree of innervation after MI have shown that animals modified in such a way are more prone to VF (3, 54). However, limited information is available about the finer structure and extent of hyperinnervation, and it is not clear whether the artificial upregulation of innervation represents real physiology or pathophysiology. Also, the fact that excess of a factor is proarrhythmic does not imply that that the reduction of the factor below its physiological level is antiarrhythmic. The proarrhythmic effects of excessive postinfarction sympathetic activation have also been suggested based on perfusion of infarcted hearts with a β-AR agonist (55). However, infarct BZs are not only differentially innervated compared with the normal myocardium but also hypoperfused. The BZ myocardium has a clearly diminished vasculature and O_2_ delivery compared with the normal myocardium (9), and wall thickening in blood vessels in the BZ may further limit perfusion (25). Thus, perfusion of a heart with heterogeneous vascularization with β-AR agonists may have pronounced heterogeneous effects that are not in agreement with heterogeneity of innervation.

Ultimately, β-blockers are a well-established antiarrhythmic treatment post-MI. However, this antiarrhythmic effect may not be due to the blockade of BZ hyperinnervation but may be due to reduced heart rate (which would also act against alternans). Furthermore, β-blockers can, surprisingly, increase cardiac innervation density (6) and, in congestive heart failure, improve sensitivity to β-AR stimulation (21). One possible antiarrhythmic mechanism may be a reduction in heterogeneity of β-AR stimulation due to these effects. On the other hand, sympathetic denervation post-MI in patients has been associated with increased incidence of arrhythmias and SCD independent of infarct size and left ventricular ejection fraction (2, 13). Similarly, restoration of cardiac sympathetic innervation in the BZ prevented arrhythmia in mice (16). Taken together, these results and the experimental data on the effects of β-AR stimulation on alternans suggest that heterogeneous innervation, resulting either from local denervation or excessive local hyperinnervation, but not sympathetic stimulation per se, is proarrhythmic and that a homogeneous re/hyperinnervation may be antiarrhythmic. In agreement, our multicellular simulations indicate that homogeneous β-AR stimulation abolishes alternans.

#### Limitations and future perspectives.

We used deterministic common pool models of the canine and human ventricular cardiomyocyte to study the effects of β-AR stimulation on alternans. Exploiting the perfect control provided by computational modeling, we identified a number of mechanisms through which β-AR stimulation reduces the likelihood of CaT alternans. However, subcellular cardiomyocyte Ca^2+^ handling is spatially heterogeneous and subject to strong local positive feedback systems. Previous work has shown that stochastic channel gating and local Ca^2+^ handling produce complex temporal repolarization variability (20, 44, 45), and these factors may similarly modulate the susceptibility to CaT alternans (46, 57). Of note, BZ cardiomyocytes undergo pronounced structural remodeling, which may further modulate subcellular Ca^2+^ handling and alternans formation. In agreement, simulated T-tubule disruption has been shown to alter the SR release/load relationship and synchronization of sparks, thereby promoting alternans (37). Here, we investigated the effects of β-AR stimulation at steady state. Hammer et al. (18) identified a transient period during β-AR stimulation in which alternans was spatially discordant. Modeling studies have shown that differences in the phosphorylation rate of individual β-AR targets may indeed result in transient periods of repolarization alternans (59), although Ca^2+^ handling was not investigated. Finally, recent tissue-level models have investigated spatial aspects of repolarization alternans in heart failure and atrial fibrillation (1, 4). Integration of the detailed β-AR signaling pathway used here in single cell and strand simulations into these tissue models and simulation of the timeframes required for β-AR stimulation currently requires a prohibitive amount of computational resources but, in the future, may enable a more detailed analysis of the effects of spatially heterogeneous sympathetic innervation on arrhythmogenesis.

#### Conclusions.

The surviving BZ myocardium surrounding an infarct is more susceptible to the development of repolarization alternans, which is primarily driven by beat-to-beat fluctuations in Ca^2+^ cycling. β-AR stimulation abolishes alternans in a computational model predominantly through direct modulation of RyR2 properties. Together, these findings suggest that BZ hyperinnervation may antagonize the development of proarrhythmic repolarization alternans.

## GRANTS

B. Rodriguez was supported by a Wellcome Trust Senior Research Fellowship in Basic Biomedical Science (100246/Z/12/Z), the British Heart Foundation Centre of Research Excellence in Oxford (RE/13/1/30181), an NC3R Infrastructure for Impact award (NC/P001076/1), an Engineering and Physical Sciences Research Council Impact Acceleration Award (EP/K503769/1), and the ComBioMed project funded by the European Union’s Horizon 2020 research and innovation program (Grant Agreement 675451). J. Heijman was supported by the Netherlands Organization for Scientific Research (NWO/ZonMW Veni 91616057).

## DISCLOSURES

No conflicts of interest, financial or otherwise, are declared by the author(s).

## AUTHOR CONTRIBUTIONS

J.T., B.R., G.B., and J.H. conceived and designed research; J.T. and J.H. performed experiments; J.T., B.R., G.B., and J.H. analyzed data; J.T., B.R., G.B., and J.H. interpreted results of experiments; J.T. and J.H. prepared figures; J.T. and J.H. drafted manuscript; J.T., B.R., G.B., and J.H. edited and revised manuscript; J.T., B.R., G.B., and J.H. approved final version of manuscript.

## Supplementary Material

Main_Model_Code.txt

Example_code_multicellular_simulation.txt

Example_code_single_cell_simulation.txt

## Figures and Tables

**Fig. A1.**
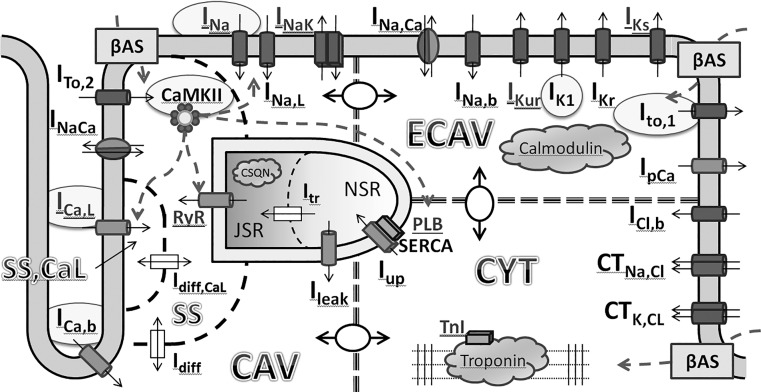
Currents and sites affected by β-adrenergic stimulation are given in gray font. Components on the background of a gray ellipse and/or circle are remodeled under conditions representing the infarct border zone.

**Fig. A2.**
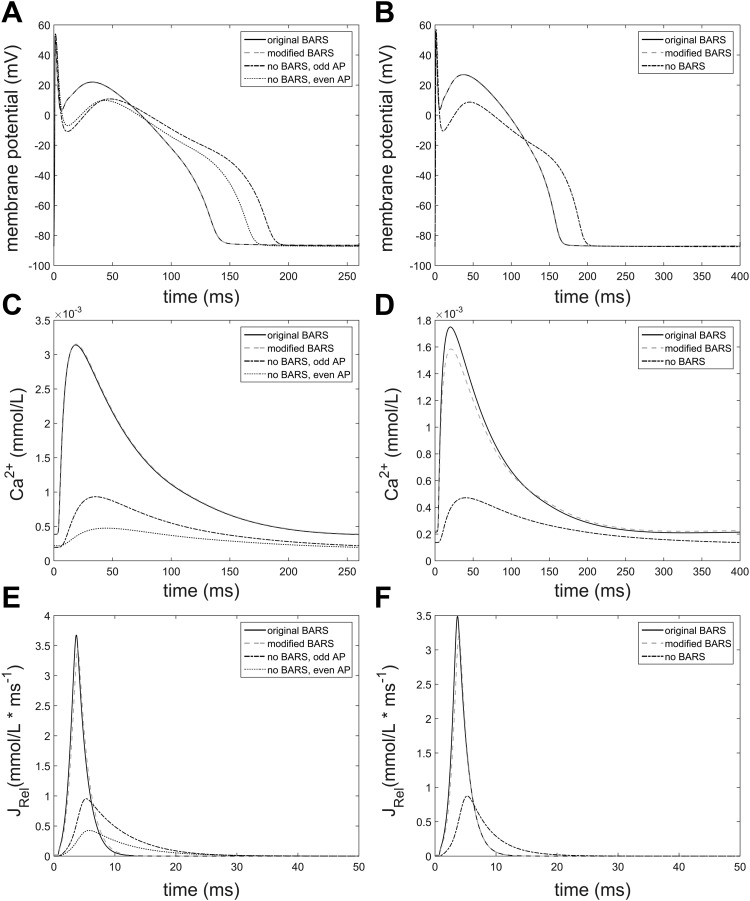
Comparison of the original and modified model of β-AR stimulation-dependent RyR2 regulation in a NZ cell, also showing traces from a NZ cell without β-AR stimulation. *A*, *C*, and *E* contain traces of membrane potential, free intracellular Ca^2+^, and Ca^2+^ release flux from the SR (having different *x*-axis) in a cell paced at a BCL of 260 ms. As the cell without β-AR stimulation manifests alternans, both alternating beats are shown. *B*, *D*, and *F* show the same variables but at a BCL of 400 ms, when no alternans is present.

**Fig. A3.**
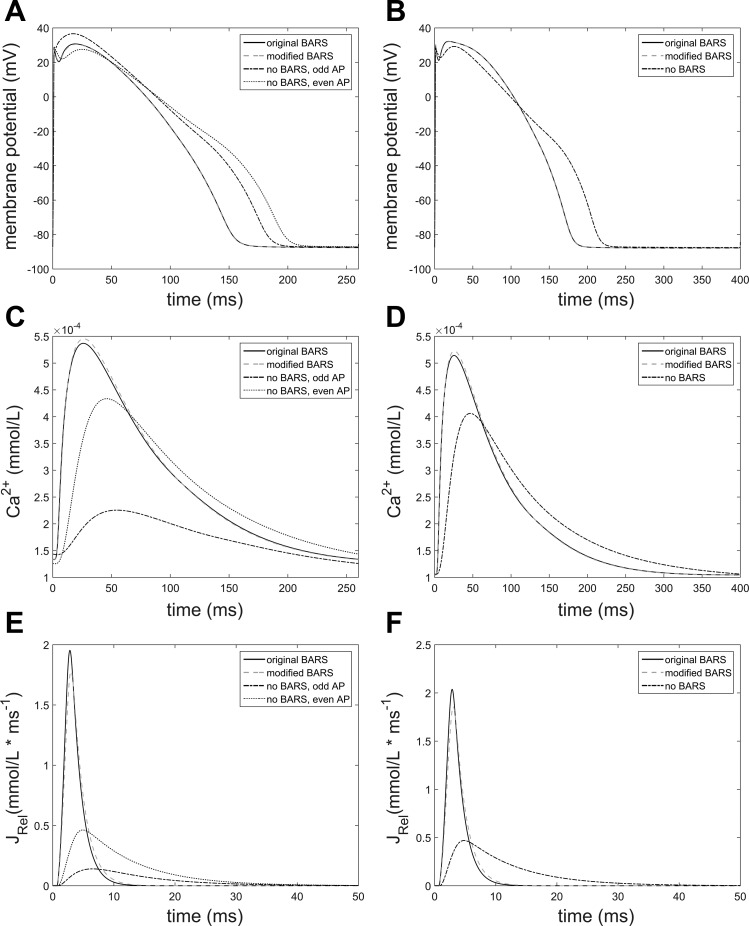
Comparison of the original and modified model of β-AR stimulation-dependent RyR2 regulation in a BZ cell, also showing traces from a BZ cell without β-AR stimulation. *A*, *C*, and *E* contain traces of membrane potential, free intracellular Ca^2+^, and Ca^2+^ release flux from the SR (having different *x*-axis) in a cell paced at a BCL of 260 ms. As the cell without β-AR stimulation manifests alternans, both alternating AP phenotypes are shown. *B*, *D*, and *F* show the same variables but at a BCL of 400 ms.

**Fig. A4.**
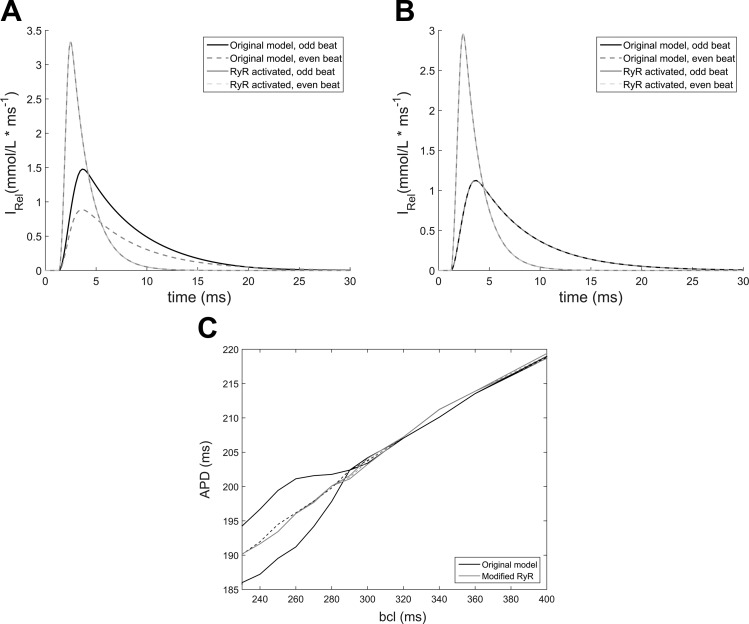
SR Ca^2+^ release in the original versus RyR2-modified human ventricular cardiomyocyte model at a BCL of 260 ms (*A*) and 400 ms (*B*). In both cases, even and odd beats are shown to facilitate the evaluation of presence of alternans. *C*: minimum and maximum APD of both cell types at each tested BCL (with average shown with dashed line). Alternans with amplitude larger than 1 ms was present between BCLs of 230 and 280 ms in the original model only. In both models, a 2:1 block occurred at BCLs below 230 ms.

## Footnotes

1 The slight difference is due to the NSR contents becoming comparatively smaller over time compared with C1 due to the lack of PLB phosphorylation which increases the reuptake and thus NSR contents.

## APPENDIX A: MODEL DIAGRAM

A diagram of the model is shown in Fig. A1.

## APPENDIX B: MODIFIED β-AR STIMULATION-DEPENDENT REGULATION OF RyR ACTIVATION

Regulation of intracellular Ca^2+^ handling is a major component of β-AR stimulation. CaT amplitude is significantly increased and upstroke and decay are considerably faster under β-AR stimulation (29, 33, 39). These features are replicated by the original model by Heijman et al. (19) as well as by our adapted model (Fig. 2). In addition, the dynamics of Ca^2+^ release from the SR through RyR2 channels are also altered, independent of SR loading and L-type Ca^2+^ current (17). In contrast, the total amount of Ca^2+^ released from the SR for a given SR Ca^2+^ load and trigger amplitude appears to be similar in the absence or presence of β-AR stimulation (17). During β-AR stimulation, RyR2 has been shown to *1*) open earlier during an AP, causing increased CaT upstroke velocity, and *2*) open more synchronously, increasing peak SR Ca^2+^ release and also shortening the total release duration (50). These macroscopic features are replicated by the phenomenological model of SR Ca^2+^ release by Heijman et al. (19). The alteration of RyR2 dynamics in the source model is implemented via *1*) explicitly increased SR Ca^2+^ leak, *2*) a reduced time constant of RyR2 channel activation and deactivation, *3*) ignoring the effect of CaMKII phosphorylation in RyR2 modulated by β-AR stimulation, and *4*) increasing the flux through RyR2 to compensate for the other changes. The additional effect of CaMKII in the phosphorylated group was removed to implicitly reflect the possibility that the β-AR stimulation-dependent changes in SR Ca^2+^ release were in fact already CaMKII mediated. In this case, further active modulation by CaMKII would not be possible. However, although there is extensive cross talk between the signaling pathways, it is generally accepted that RyR2 has separate phosphorylation sites for β-AR stimulation-activated PKA (Ser^2809^ and/or Ser^2030^) and CaMKII (Ser^2815^). Nonetheless, the exact functional effects of PKA- and CaMKII-dependent RyR2 phosphorylation remain a topic of active debate.

To simplify the model and generalize its structure to allow independent regulation by both PKA and CaMKII, we developed an alternative model for SR Ca^2+^ release with three β-AR stimulation-dependent parameters and similar CaMKII effects on both phosphorylated and nonphosphorylated RyR2. We found that a near-identical phenotype to the original model can be obtained by maintaining the explicitly increased SR leak, setting the multiplier of SR release (RyR2_Amp) to 0.65 (original value: 1.9925), and reducing the multiplier of time constant of RyR2 opening and closing to 40% of its original value of 0.5357. Figure A2 shows a comparison of the original and modified model of β-AR stimulation-dependent RyR2 regulation in the NZ model under maximal β-AR stimulation (1.0 µmol/l ISO), and the traces were compared with the case of no β-AR stimulation. It should be noted that we have only changed the β-AR stimulation-dependent regulation of RyR2 and that SR Ca^2+^ release in the absence of β-AR stimulation is therefore unaffected. A similar comparison was performed in BZ cells, as shown in Fig. A3. Figures A2 and A3 show that the modified model behaves similarly to the original model at pacing frequencies relevant to the research of alternans formation.

## APPENDIX C: SIMULATING THE EFFECTS OF RyR2 ACTIVATION BY β-AR STIMULATION IN THE O’HARA-RUDY HUMAN VENTRICULAR CARDIOMYOCYTE MODEL

To validate the importance of altered SR Ca^2+^ release dynamics in attenuating alternans observed in the Heijman et al. (19) canine ventricular cardiomyocyte model, we implemented a similar RyR2 activation in the state-of-the-art human ventricular cardiomyocyte model by O’Hara et al. (38). The endocardial parametrization of the model has been used, as it was the only one that manifested alternans at rapid pacing rates. To achieve the faster activation and shorter duration of SR Ca^2+^ release during β-AR stimulation-dependent RyR2 activation, we reduced the activation constant (β_τ_) to 1.583 ms (1/3 of the original value of 4.75 ms) to represent faster RyR2 opening and closing, and we increased the modifier of SR Ca^2+^ release amplitude six times to match the total amount of Ca^2+^ released from SR to a cell with unchanged RyR2 dynamics. The revised model indeed manifests a shorter Ca^2+^ release of increased amplitude (Fig. A4, *A* and *B*), consistent with the experimentally observed effects of β-AR stimulation-dependent RyR2 regulation (17).

With these modifications, the integral of release per two APs at a BCL of 260 ms in a normal cell was 16.69 versus 17.16 in the revised model incorporating β-AR stimulation-dependent RyR2 regulation. At a BCL of 400 ms, the integral over two APs was 15.89 in a normal cell and 15.3 in a RyR2-activated cell, respectively, showing that at pacing frequencies relevant for alternans formation, the altered SR Ca^2+^ release dynamics did not considerably change the total amount of Ca^2+^ released. As shown in Fig. A4*C*, the original human ventricular cardiomyocyte model manifested alternans at a BCL smaller than 280 ms, whereas the model with altered RyR2 dynamics never exceeded alternans amplitudes of 1 ms, in agreement with our findings in the canine model.

## APPENDIX D: MODEL STABILITY DEFINITION

In an oscillatory system possibly manifesting alternans, the notion of stability needed to be redefined compared with the common definition. We consider the cellular model to be in quasistable state when the maximum and minimum value of a variable (*var*) during two consecutive beats each change <0.2% per 100 beats for *var = *membrane potential, intracellular Ca^2+^, intracellular K^+^, and intracellular Na^+^. Maxima and minima of beat pairs are used to allow cells manifesting alternans to be considered stabilized. For all tested pacing frequencies, 2,500 beats were sufficient to reach the quasistable state.
